# From valve prosthesis to coronary artery: a fatal thrombus: a case report

**DOI:** 10.1093/ehjcr/ytag070

**Published:** 2026-03-02

**Authors:** Carlos Cobo-Lopez, Gloria Rocio Padilla-Rodriguez, Alejandro Gomez-Gonzalez, Francisco Javier Cortes-Cortes, Manuel Almendro-Delia

**Affiliations:** Cardiology Unit, University Hospital Virgen Macarena, Avda Doctor Fedriani, 3, 41009 Sevilla, Spain; Cardiology Unit, University Hospital Virgen Macarena, Avda Doctor Fedriani, 3, 41009 Sevilla, Spain; Acute Cardiovascular Care Unit, University Hospital of the Canary Islands, Carretera Ofra S/N, Carr. Gral. la Cuesta, 79, 38320 La Laguna, Santa Cruz de Tenerife, Spain; Cardiology Unit, University Hospital Virgen Macarena, Avda Doctor Fedriani, 3, 41009 Sevilla, Spain; Cardiology Unit, University Hospital Virgen Macarena, Avda Doctor Fedriani, 3, 41009 Sevilla, Spain

**Keywords:** Acute myocardial infarction, Coronary embolism, Anticoagulation, Mechanical valve prostheses, Case report

## Abstract

**Background:**

Coronary embolism is an uncommon cause of acute myocardial infarction, often related to atrial fibrillation or valvular heart disease. In such cases, concomitant valvular thrombosis requires additional diagnostic work-up and has major implications for subsequent management. Both conditions may represent significant diagnostic and therapeutic challenges.

**Case summary:**

We report the case of a 71-year-old woman with double mechanical valve prostheses (aortic and mitral) under suboptimal anticoagulation therapy, who presented with chest pain and syncope. Initial workup revealed severe right ventricular dysfunction and dilation, with inconclusive electrocardiogram findings; pulmonary embolism was initially suspected. Emergent coronary angiography revealed a coronary embolism, successfully treated with thromboaspiration alone. The diagnosis of prosthetic valve thrombosis was delayed due to low cardiac output masking prosthetic dysfunction. Management of the valve thrombosis was unconventional, as therapeutic options were limited by a recent traumatic brain injury.

**Discussion:**

This case illustrates the diagnostic and therapeutic challenges in patients with mechanical valve prostheses and high embolic risk. Atypical presentation and low-output conditions limited early identification of prosthetic valve thrombosis, highlighting the reduced sensitivity of transthoracic echocardiography in this context. Advanced imaging modalities, such as transoesophageal echocardiography or computed tomography, may improve diagnostic accuracy. Given absolute contraindications to both thrombolysis and surgery, the patient was managed with intensified anticoagulation, showing favourable clinical and echocardiographic evolution. This case emphasizes the need for individualized, context-driven decision-making in complex cardiovascular scenarios.

Learning points

- Coronary embolism should be suspected in STEMI patients with mechanical heart valves.
- In low-output states, transvalvular gradients may be underestimated, making TEE and cardiac CT essential for diagnosing prosthetic valve thrombosis.
- In the management of prosthetic valve thrombosis, when thrombolysis or surgery is contraindicated, intensified anticoagulation is a valid alternative.

## Introduction

Coronary embolism is a rare cause of acute myocardial infarction (AMI), accounting for 3–4% of cases, with prosthetic valve thrombosis being one of the potential aetiologies. We present the case of a frail female patient with a history of rheumatic heart disease, double mechanical valve replacement, and a permanent pacemaker, who was admitted for embolic ST-elevation myocardial infarction (STEMI) and cardiogenic shock.

## Summary figure

**Figure:**
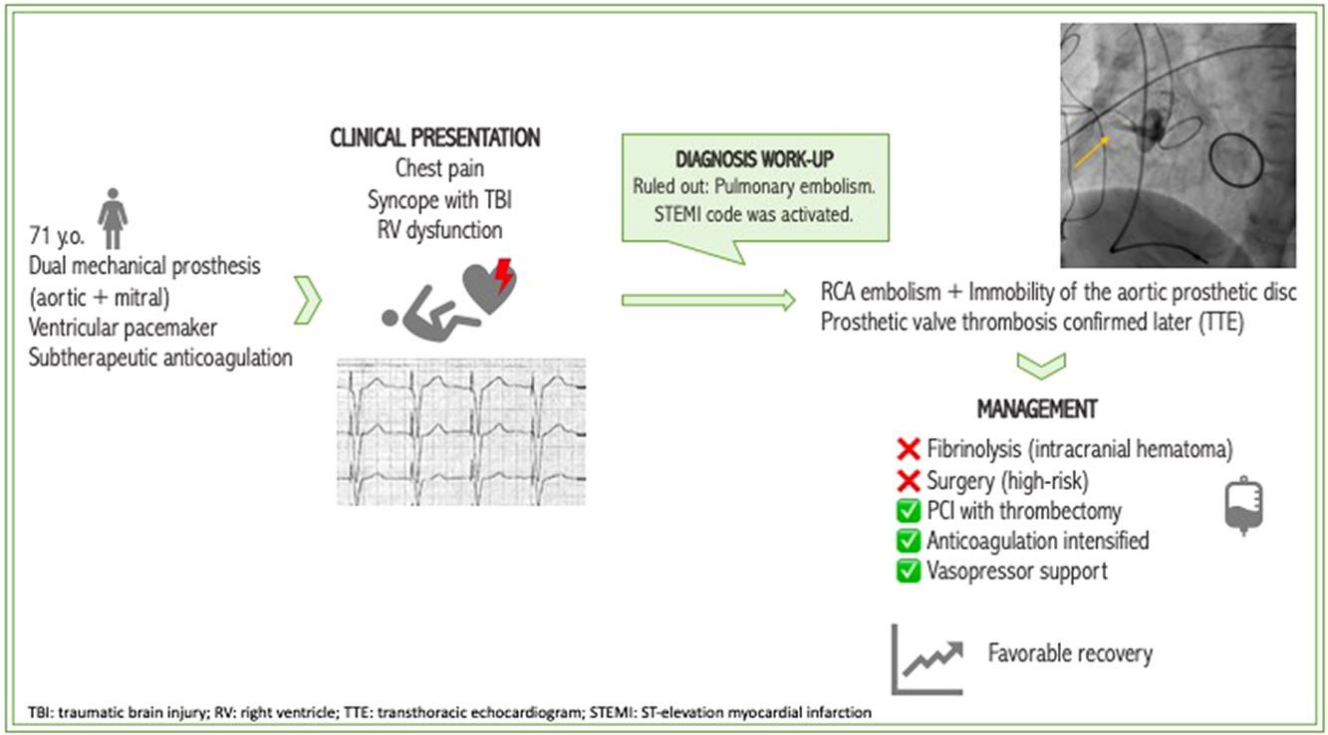


## Case presentation

A 71-year-old woman with a low body mass index (BMI 17.3 kg/m^2^), a former smoker, with dyslipidaemia and hypertension. Her medical history included aortic (St. Jude no21) and mitral (St. Jude no27) mechanical valve replacement in 2014 due to rheumatic heart disease. A follow-up transthoracic echocardiogram (TTE) in 2023 showed a mean transaortic gradient (MG) of 11.3 mmHg, transmitral MG of 1.25 mmHg, and normal biventricular function and chamber volumes. She had permanent atrial fibrillation, a VVI pacemaker, and atrioventricular node ablation in 2023. Other comorbidities included embolic right hemispheric transient ischaemic attack, GOLD 1 Chronic obstructive pulmonary disease, severe osteoporosis, and sarcopenia. Medications included acenocoumarol (with poor INR control), atorvastatin 40 mg, empagliflozin 10 mg, bisoprolol 2.5 mg, esomeprazole 20 mg, tiotropium bromide, furosemide 40 mg, subcutaneous teriparatide 20 µg, and calcifediol 266 µg.

She presented with one hour of oppressive chest pain followed by sudden syncope with immediate recovery, along with associated traumatic brain injury (TBI). On physical examination: Hypotension (BP 75/45 mmHg), heart rate 70 bpm, preserved consciousness, pallor, and distal coldness. Auscultation revealed prosthetic valve clicks without murmurs or pulmonary rales. No peripheral oedema.

**Figure 1:**
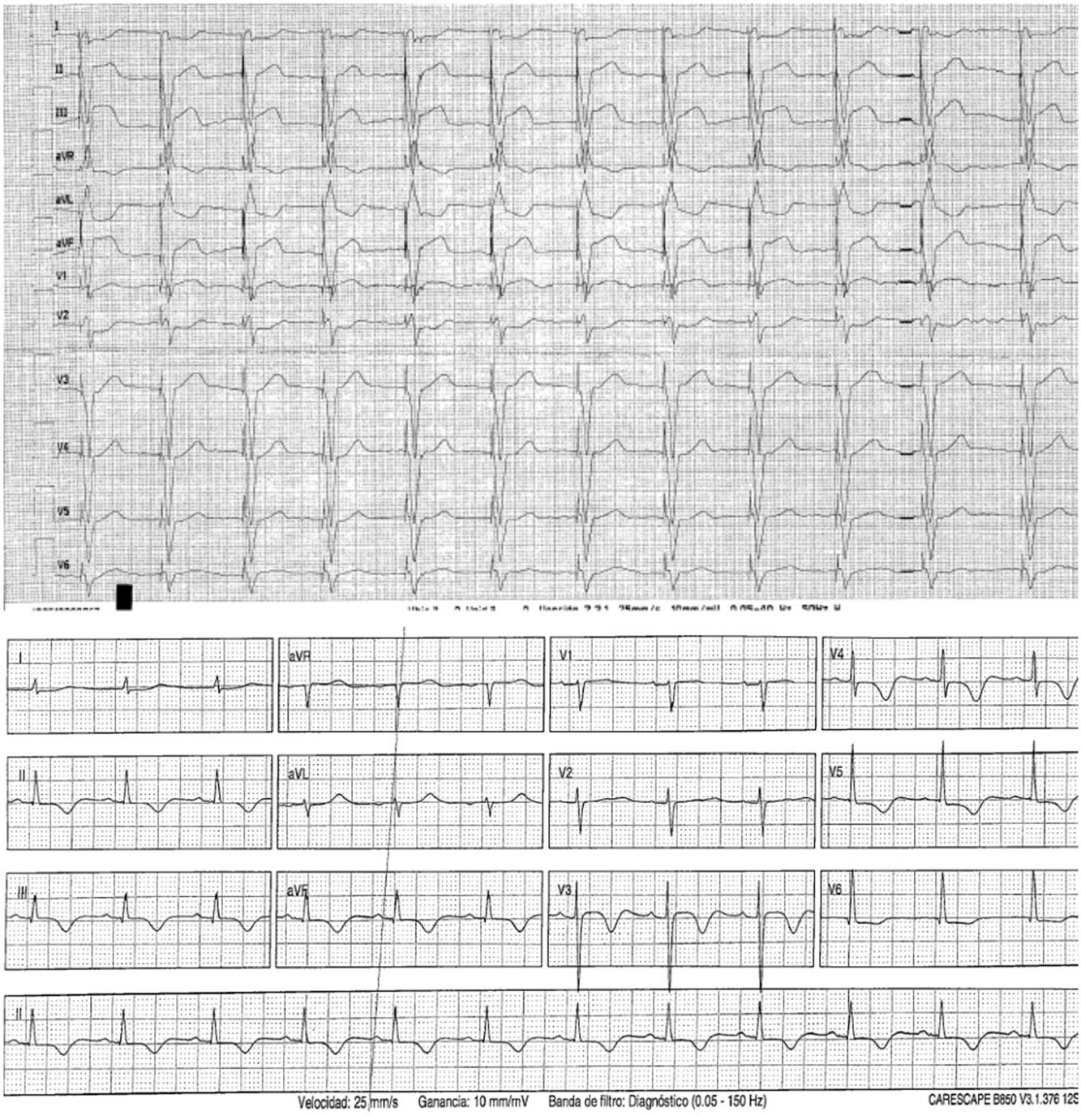
Electrocardiograms. On admission (ventricular pacing), after coronary reperfusion (intrinsic rhythm).

Initial ECG showed ventricular pacing at 70 bpm, without Sgarbossa criteria (*Figure 1*). Lab tests showed: troponin I 908 ng/mL, haemoglobin 15.1 g/dL, INR 2.47, aPTT 40.6 s, creatinine 0.78 mg/dL, AST 277 U/L, ALT 253 U/L, and lactic acid 4.5 mmol/L.

**Figure 2:**
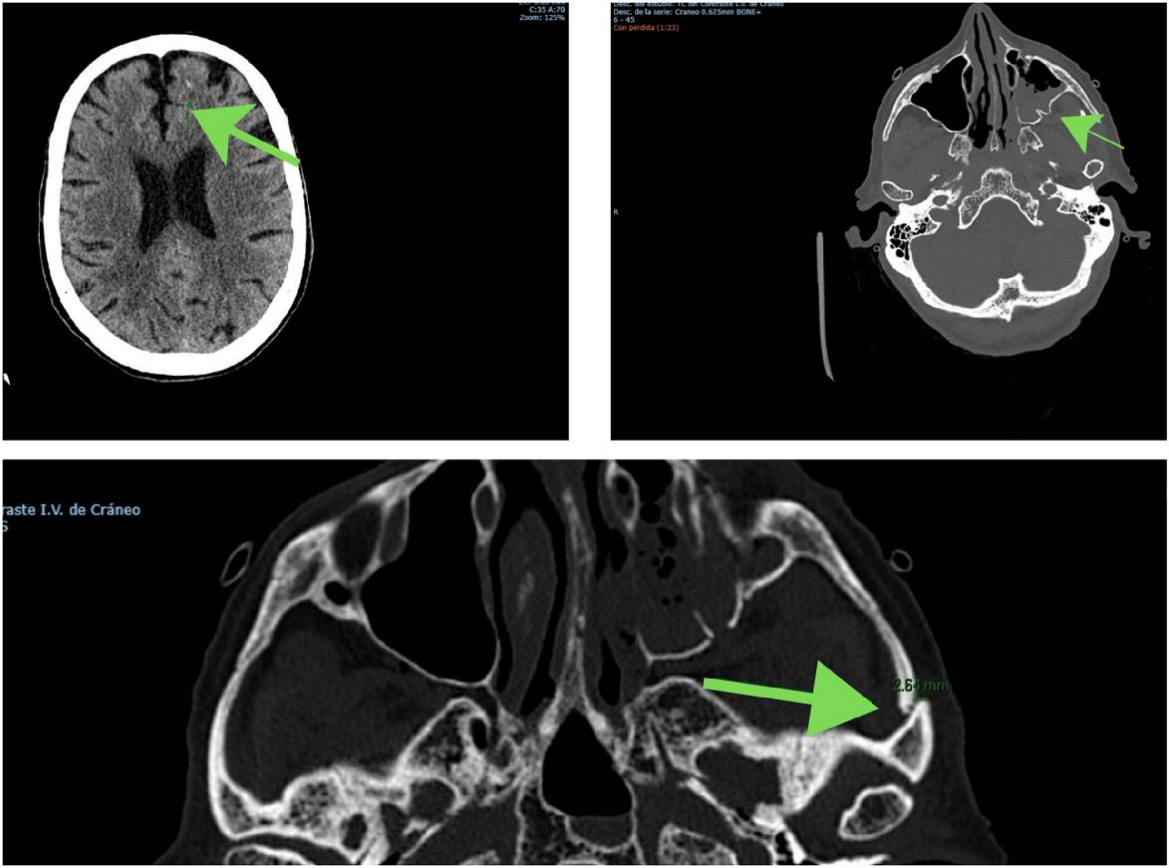
Brain computed tomography: left frontal contusion and fracture of the lateral wall and floor of the left orbit with bone fragment displacement.

Bedside TTE revealed severe right ventricular (RV) dilation and dysfunction. A chest CT angiogram ruled out pulmonary embolism. Brain CT showed a left frontal contusion and fracture of the lateral wall and floor of the left orbit with bone fragment displacement (*Figure 2*).

**Figure 3:**
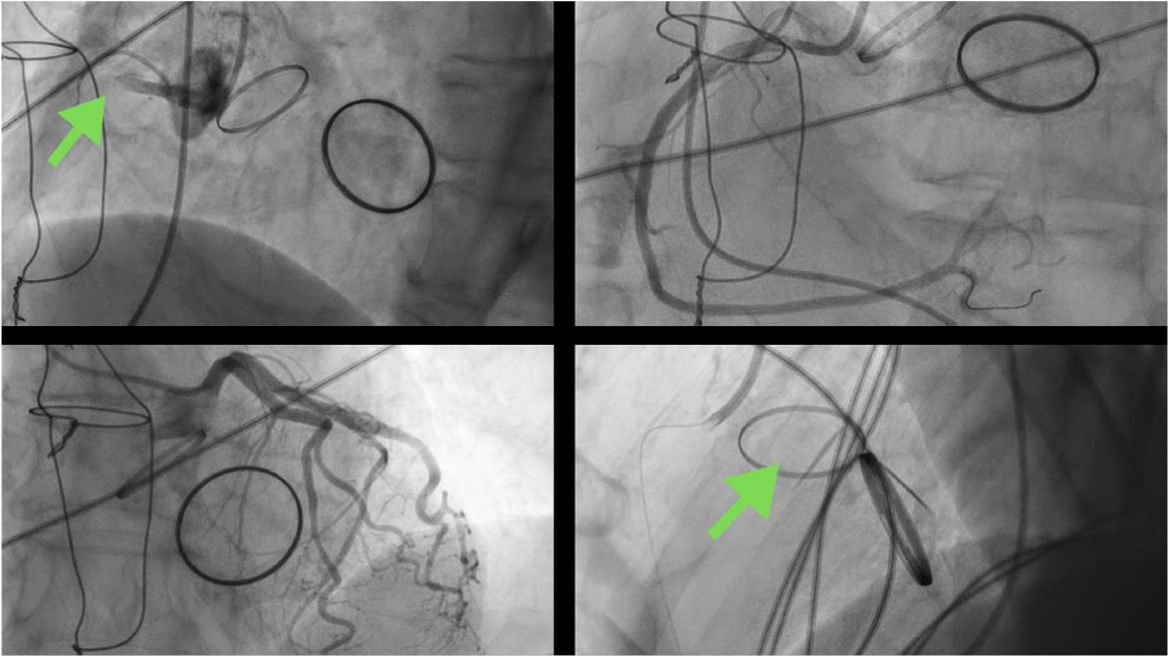
Coronary angiography. Top: Occlusion of the right coronary artery and the reperfused vessel. Bottom: View of the valvular prostheses showing only one mobile disc in the aortic prosthesis.

Due to persistent haemodynamic instability and chest pain, the STEMI protocol was activated. Coronary angiography revealed a thrombotic occlusion of the proximal right coronary artery (RCA), which was successfully recanalized by thrombus aspiration, with no evidence of atherosclerotic plaques. Fluoroscopy showed the absence of movement of one of the disks of the aortic prosthesis, raising suspicion of prosthetic valve thrombosis (PVT) (*Figure 3*). Treatment was initiated with aspirin, clopidogrel, and intravenous unfractionated heparin (UFH).

Post-revascularization TTE showed normal left ventricular (LV) systolic function, severely dilated RV (basal diameter 60 mm), RV dysfunction (TAPSE 12 mm, S’ wave 6 cm/s), and severe tricuspid regurgitation. Stroke volume was low (LVOT VTI 11 cm), and transprosthetic gradients were similar to baseline, with no visible thrombus. Transoesophageal echocardiography (TEE) was indicated but deferred due to haemodynamic instability.

In the first days of admission, she required norepinephrine and dobutamine at moderate doses for perfusion support, maintaining normal lactate levels and adequate peripheral perfusion. Despite prior AV node ablation, an intrinsic rhythm was observed on monitoring, so pacing was reduced to 50 bpm to enhance interventricular synchrony.

She experienced bleeding from vascular access sites due to elevated INR, leading to temporary discontinuation of anticoagulation until a repeat brain CT ruled out haemorrhagic progression. Once INR normalized, anticoagulation was resumed with low molecular weight heparin (LMWH), adjusted by anti-Xa levels. Clopidogrel was discontinued due to the absence of coronary atherosclerosis, to minimize bleeding risk.

**Figure 4:**
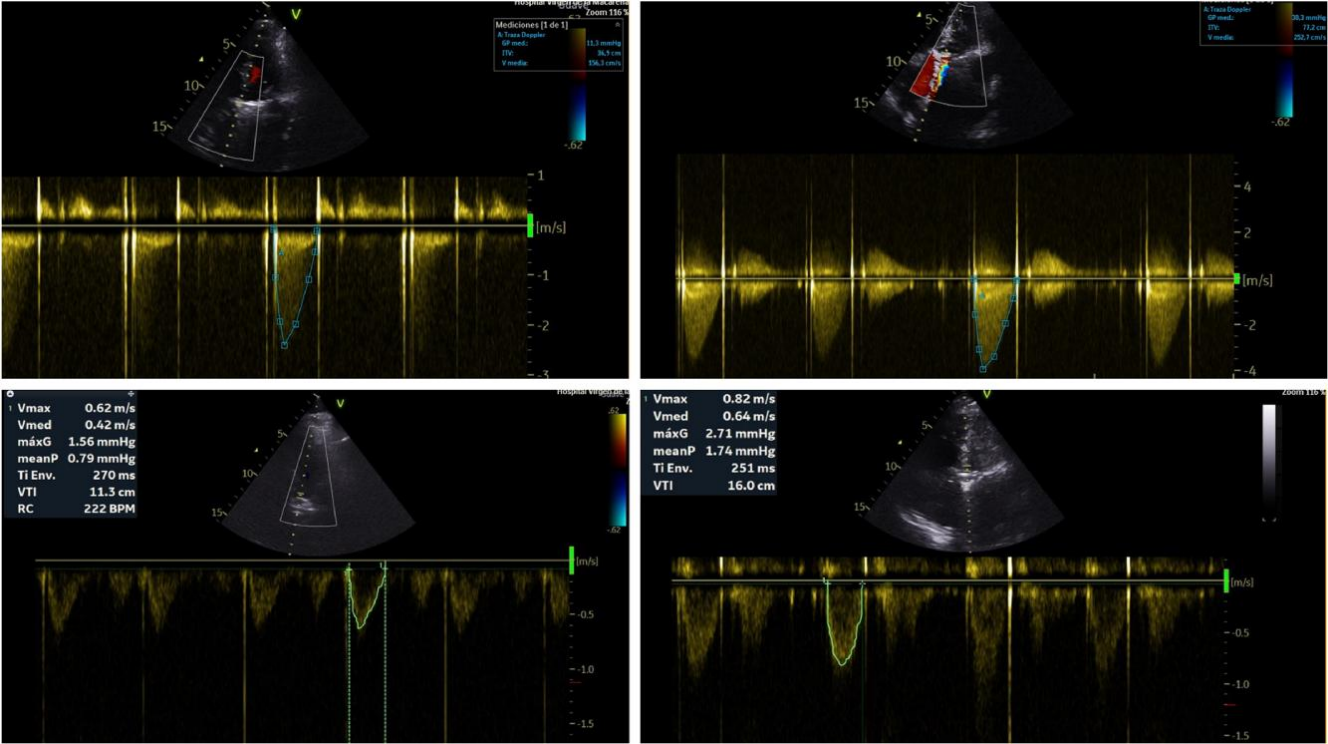
Continuous-wave Doppler. Trend of cardiac output and transaortic gradient.

Follow-up echocardiograms showed improved cardiac output (LVOT VTI 16 cm) and a progressive increase in aortic MG (up to 36 mmHg) (*Figure 4*), prompting TEE, which revealed a thrombus on the anterior disk of the aortic prosthesis. UFH was restarted with aPTT monitoring.

Cardiac surgery was consulted, but given progressive haemodynamic improvement and persistent high surgical risk (RV dysfunction, frailty, and intracranial haemorrhage), surgery was deferred. Repeat brain CT showed a 3 mm left subdural haematoma.

She was transferred to a medical ward, where UFH was replaced with LMWH. She required rehabilitation due to critical illness polyneuropathy and nutritional support. Maxillofacial and ophthalmology teams recommended conservative management of facial injuries due to the TBI.

A final TEE showed no visible thrombus and a slight reduction in transprosthetic gradients. A multidisciplinary team opted for definitive medical management. As the intrinsic rhythm persisted, the pacing rate was maintained at 50 bpm.

In the final days of hospitalization, the patient progressively improved and began ambulation. Oral anticoagulation with warfarin was restarted with close INR monitoring.

At 3-month outpatient follow-up, she remains clinically stable with no signs of heart failure, non-obstructive transprosthetic gradients, and stable INR control.

## Discussion

Prosthetic valve thrombosis (PVT) is a rare but potentially life-threatening complication, which may present as either obstructive valve dysfunction or embolic events. Coronary embolism accounts for approximately 3–4% of all AMI cases, and prosthetic valve thrombosis is a recognized but uncommon aetiology.^1^

This case presented multiple diagnostic and therapeutic challenges. The patient, with double mechanical valve prostheses and suboptimal anticoagulation, presented with chest pain, syncope likely of cardiac origin, and severe RV dilation and dysfunction. The presence of a ventricular pacemaker hindered ECG interpretation by masking typical STEMI findings. The initial clinical and haemodynamic profile suggested pulmonary embolism, delaying the correct diagnosis of embolic STEMI.

Emergent coronary angiography revealed thrombotic occlusion of the RCA, treated successfully with thrombus aspiration, without the need for stent implantation. Although the angiographic outcome was satisfactory, intracoronary imaging (OCT, IVUS) could have provided greater certainty regarding the absence of atherosclerosis, as reported in similar cases.^2,3^

Initial suspicion of aortic prosthesis thrombosis arose from the absence of disk motion on fluoroscopy, although the first TTE showed no elevated gradients, likely due to low cardiac output. As the patient improved, rising transprosthetic gradients confirmed the diagnosis. TEE and CT offer greater sensitivity in low-flow states for detecting thrombus, disk immobility, or attached masses.

Management options for mechanical PVT include intensified anticoagulation, thrombolysis, and surgery. ESC (2025) and AHA/ACC (2020) guidelines recommend considering thrombolysis in selected patients without contraindications, particularly when surgery is high-risk or unavailable.^4,5^ Low-dose slow-infusion thrombolysis protocols, such as HATTUSHA, have shown success rates over 80%.^6^

However, thrombolysis was absolutely contraindicated in this case due to traumatic brain injury and intracranial haematoma. Surgery was also ruled out given the patient’s high operative risk (RV dysfunction, frailty, and intracranial bleeding). Therefore, management consisted of UFH-based anticoagulation following coronary reperfusion. Anticoagulation control was challenging due to the patient’s low BMI, malnutrition, and high bleeding risk. Despite this, clinical and echocardiographic evolution was favourable.

This case underscores the importance of individualized management based on comprehensive clinical assessment and patient-specific risks.

## Data Availability

Further echocardiography and CT images are available from the corresponding author.

## Lead author biography

Cardiology resident at Virgen Macarena University Hospital, Seville

## Notes

**Consent:** We have received the patient's informed consent for the use of data from their medical records and clinical images obtained for publication, in accordance with COPE guidelines.
